# A data-driven approach to decompose motion data into task-relevant and task-irrelevant components in categorical outcome

**DOI:** 10.1038/s41598-020-59257-z

**Published:** 2020-02-12

**Authors:** Daisuke Furuki, Ken Takiyama

**Affiliations:** Department of Electrical and Electronic Engineering, Tokyo University of Agriculture and Technology, Koganei-shi, Tokyo 184-8588 Japan

**Keywords:** Motor control, Sensorimotor processing

## Abstract

Decomposition of motion data into task-relevant and task-irrelevant components is an effective way to clarify the diverse features involved in motor control and learning. Several previous methods have succeeded in this type of decomposition while focusing on the clear relation of motion to both a specific goal and a continuous outcome, such as a 10 mm deviation from a target or 1 m/s hand velocity. In daily life, it is vital to quantify not only continuous but also categorical outcomes. For example, in baseball, batters must judge whether the opposing pitcher will throw a fastball or a breaking ball; tennis players must decide whether an opposing player will serve out wide or down the middle. However, few methods have focused on quantifying categorical outcome; thus, how to decompose motion data into task-relevant and task-irrelevant components when the outcome is categorical rather than continuous remains unclear. Here, we propose a data-driven method to decompose motion data into task-relevant and task-irrelevant components when the outcome takes categorical values. We applied our method to experimental data where subjects were required to throw fastballs or breaking balls with a similar form. Our data-driven approach can be applied to the unclear relation between motion and outcome, and the relation can be estimated in a data-driven manner. Furthermore, our method can successfully evaluate how the task-relevant components are modulated depending on the task requirements.

## Introduction

Diverse features of motor control have been reported. One striking feature is that motion data can be decomposed into at least two components: task-relevant and task-irrelevant components. Variability in task-relevant components is smaller than in task-irrelevant components^1^. Quantifying task-relevant and task-irrelevant components can thus reveal the salient features of motor control.

Several methods can quantify task-relevant and task-irrelevant motion components. The uncontrolled manifold (UCM) method quantifies the components by focusing on forward kinematics, such as hand position in sit-to-stand^2^. While the UCM focuses on kinematic outcome (e.g., the position of hand or center of mass), the goal equivalent manifold (GEM)^3^ and noise-tolerant-covariance (TNC)^4^ methods quantify the task-relevant and task-irrelevant motion components by explicitly defining the relations between kinematic parameters and task outcome (e.g., in ball throwing, the height of the ball flight can be approximated by parabolic motion). In addition to the GEM and the TNC, our recent methods detect the task-relevant and task-irrelevant motion components after estimating the unknown relation between time-varying motion and task outcome in a data-driven manner^5,6^.

Although these methods succeeded at quantifying task-relevant and task-irrelevant components, they all focused on continuous task outcomes, such as the error between desired and actual outcome^3,4,6^ or hand position^2^. In contrast, task-relevant and task-irrelevant motion components are more difficult to quantify when the outcomes are categorical, such as throwing a fastball or breaking ball, whether the action results in success or failure, serving wide or down the line in tennis, whether the subject is a healthy control or a patient, and whether the player is an amateur or professional. Despite the fact that investigating the influences of these categorical outcomes can be central topics in biomechanics, motion science or related research areas, how to decompose motion data into task-relevant and task-irrelevant components remains unclear.

In this paper, we propose a data-driven technique to detect task-relevant and task-irrelevant motion components with categorical task outcomes. Our method relies on a linear regression technique for solving classification problems, such as logistic regression^7^. For example, logistic regression enables classification of whether the current motion data are associated with throwing a fastball or breaking ball. Our data-driven method can be applied even when the relation between motion data and outcome is unknown: the relation can still be estimated in a data-driven manner. Along with recent data-driven approaches in biomechanics that focused on unsupervised methods^8,9^, we rely on supervised methods to address the task-relevant and task-irrelevant components with categorical outcomes in a data-driven manner. Notably, our current mathematical framework is the same as that used in our previous methods^5,6^, which focused on continuous task outcomes by applying a linear regression technique. Along with our previous methods, we propose a unified data-driven approach to detect task-relevant and task-irrelevant motion components for multiple kinds of task outcomes.

## Methods

### Participants

Eight healthy volunteers (aged 18-21 years, four females) participated in our experiment for two days (not consecutively). All the participants were informed of the experimental procedures and their conformance with the Declaration of Helsinki, and all participants provided written informed consent before the start of the experiments. All the procedures were approved by the Ethics Committee of the Tokyo University of Agriculture and Technology.

### Experimental protocol

**Figure 1:**
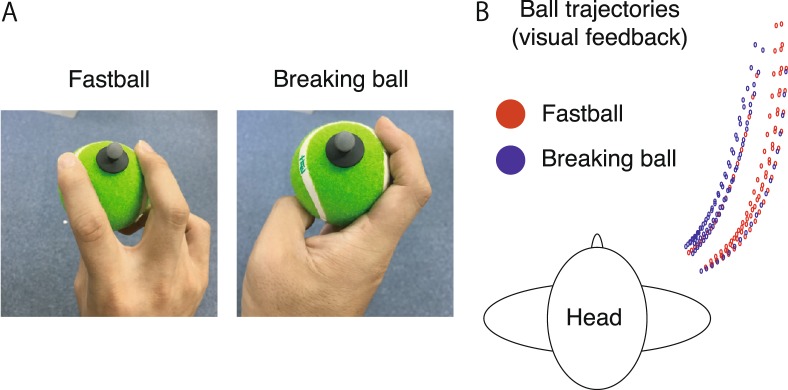
Protocol of our experiments. (**A**) The instructions for subjects described how to grasp the ball when throwing a fastball or breaking ball. (**B**) On the second day, the subjects were instructed to throw the fastballs and breaking balls using a similar form. The subjects could confirm the trajectory of the marker attached to the ball only on the second day.

The subjects were seated on a chair and instructed to throw a ball towards a target 2.8 meters from the chair. We used one target location throughout this study to focus on whether the subjects threw either a fastball or breaking ball. On the first and the second days, the subjects were instructed to throw either a fastball or breaking ball pseudorandomly (i.e., they threw each once in every two trials) towards the target following their preferred motion form. Because all the participants were naive to baseball pitching, how they grasped the ball was the only difference between throwing the two types of pitches (Fig. 1A). On the second day, the subjects were additionally instructed to throw a fastball and a breaking ball using a similar motion. The purpose of the current study is to investigate the influence of task instruction on task-relevant and task-irrelevant motion components. To facilitate similar forms, we provided feedback about the motion trajectories by displaying the ball trajectories on a monitor only on the second day (Fig. 1B, 20 frames before the release).

At the beginning of each trial, three beeps sounded at a one-second interval. The subjects were instructed to throw either a fastball or breaking ball at the timing of the third beep.  On each day, subjects performed 100 main trials (50 for fastball and 50 for breaking ball) after 20 practice trials (10 for fastball and 10 for breaking ball). We analyzed the 100 main trials.

### Data acquisition and processing

Subjects’ motions were recorded at 120 Hz using nine cameras (Optitrack Flex 13, NaturalPoint Inc., Corvallis, Oregon). Markers were attached to the ball, neck, right clavicle, right shoulder, right elbow, right wrist, right index finger, and right ilium of the participants. The marker position data were filtered in MATLAB 2016a (Mathworks, Inc) using a 12th-order, 10 Hz, and zero-phase Butterworth filter. The joint angles of the shoulder (three degrees of freedom), elbow (one degree of freedom), and wrist (three degrees of freedom) were analyzed. The release timing was determined based on the moment at which the distance between the markers attached to the ball and index finger exceeded 0.01 meter. We did not analyze the trajectory of the marker attached to the ball except to detect the release time.

### Logistic regression

We applied logistic regression to classify whether the subjects threw a fastball or breaking ball based on time-varying motion data ***X*** ∈ **R**^*T*×*D*^, where *T* and *D* denoted the number of trials to be analyzed and the dimensions of the time-varying motion data. In the current study, ***X*** corresponded to the vectorized temporal sequences of the joint angles^6^. We defined the target value ***d*** ∈ **R**^*T*×1^ as *d*_*t*_ = 0 when the subjects threw the fastball; otherwise, *d*_*t*_ = 1, (*t* = 1, . . . , *T*). In the logistic regression, appropriate weight values ***w*** ∈ **R**^*D*×1^ were estimated to predict the target value based on *p*(*d*_*t*_ = 1) = *f*(*w*_0_ + ***X***_*t*_***w***), where *p*(*d*_*t*_ = 1) denotes the estimated probability that *d*_*t*_ = 1 (i.e., *p*(*d*_*t*_ = 0) = 1 − *p*(*d*_*t*_ = 1)), $f(z)=\frac{1}{1+\exp (-z)}$ denotes a sigmoid function, and *w*_0_ is a bias term to be estimated. The motion sequence data ***X*** were normalized so that the mean and standard deviation of each component across the trials were 0 and 1, respectively, following a standard cross-validation procedure^10^ that did not affect the results^11^.

Appropriate *w*_0_ and ***w*** values were estimated to minimize the following cost function *E*:

1 $$E({w}_{0},{w})=-\frac{1}{T}{\sum }_{t=1}^{T}({d}_{t}\log f({w}_{0}+{{X}}_{t}{w})+(1-{d}_{t})\log (1-f({w}_{0}+{{X}}_{t}{w})))+\frac{\lambda }{2}{{w}}^{T}{w},$$

where ***w***^*T*^ indicates the transposition of ***w***. The first and second terms on the right-hand side indicate that the cross entropy equaled 0 when logistic regression provided a perfect prediction and log2 when the prediction failed (i.e., f = 0.5 when d = 0 and d = 1). The third term is a regularization term using the parameter *λ*. An appropriate *λ* value was determined based on 10-fold cross-validation with five iterations. We used glmnet^11^ throughout this study.

**Figure 2:**
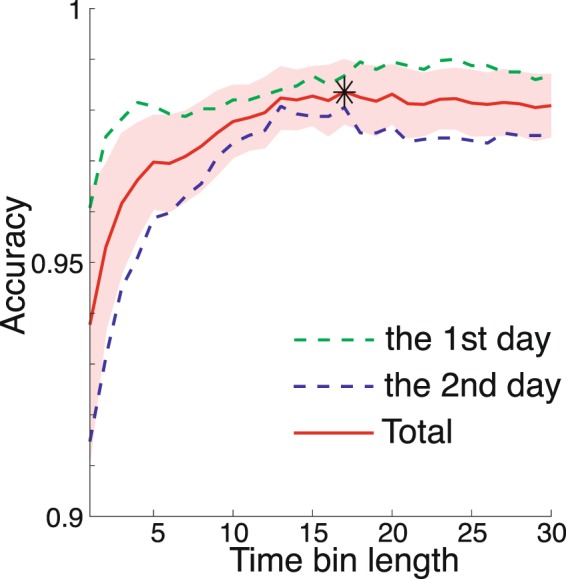
Classification outcome using logistic regression. The horizontal axis denotes the number of time frames to define time-varying motion data ***X***, and the vertical axis denotes the classification accuracy in cross-validation. The green and blue dotted lines denote the classification accuracy averaged across all subjects using the motion data on the first and second days, respectively. The red solid line and the shaded area indicate the mean and standard error of the classification accuracy for all subjects and two days, respectively. We used joint angles with 17 time frames to determine the motion sequence data ***X*** for the analysis throughout this paper because the mean classification accuracy was the best for the logistic regression with 17 time frames.

Because the relation between the time-varying motion data and task outcome is unclear in general, we confirmed whether logistic regression was able to discriminate between the motions used to throw a fastball or breaking ball. A correct classification means that the logistic regression for the motion to throw a fastball results in *p*(*d*_*t*_ = 0) > 0.5 (i.e., *p*(*d*_*t*_ = 1) < 0.5), while the motion used to throw a breaking ball results in *p*(*d*_*t*_ = 1) > 0.5 (i.e., *p*(*d*_*t*_ = 0) < 0.5). Fig. 2 shows the percentage of accurate classifications (i.e., accuracy). Because the best classification accuracy was achieved when we used 17 time frames before the release to determine ***X***: 98.37  ±  0.0063 % (mean  ±  standard error of mean [s.e.m.]), we used these 17 time frames in the following analysis. Another measure to evaluate the classification accuracy is area under the curve, or AUC. The AUC was 0.9983  ±  0.0035, indicating high classification accuracy. We focused on the time series of the seven joint angles; thus, we analyzed 119 data points for each trial.

### Statistical analysis

Repeated measures analysis of variance (ANOVA) were conducted when there were no assumptions about the statistical tests, followed by Tukey’s post hoc comparisons. The current study considered two within-subject factors, including ‘Part’ (former or latter part of the same day) and ‘Day’ (day 1 or 2). All the statistical analyses were performed using MATLAB 2016a.

## Results

Eight subjects threw fastballs and breaking balls towards a fixed target in 100 trials (50 trials for each pitch type) while seated on two days. On the first day, the subjects were instructed to throw the ball towards the target. On the second day, the subjects were additionally instructed to throw the fastball or the breaking ball with a similar motion. We analyzed seven joint angles (three shoulder joint angles, one elbow joint angle, and three wrist angles) to investigate how the task-relevant components were modulated depending on the task requirement. Throughout this study, we relied on logistic regression, although the method to extract task-relevant and task-irrelevant motion components was invariant across linear classification methods as follows.

### Decomposition into task-relevant and task-irrelevant differences in categorical outcome

Using linear classification methods such as logistic regression, we can obtain weight values ***w*** to determine the probability that the analyzed data will be classified into the first or second category (i.e., *p*(*d*_*t*_ = 1) = *f*(*w*_0_ + ***X***_*t*_***w***), where *d*_*t*_ is the target value and *d*_*t*_ = 1 indicates that the category associated with motion data at the *t*th trial, ***X***_*t*_, is the breaking ball, *f* is a sigmoid function, and *w*_0_ is the bias to be estimated). Based on these results, we can discuss how each joint angle at each time point contributes to the categorical task outcome (i.e., fastball or breaking ball in the current study)^5^. The purpose of this study was to focus on task-relevant and task-irrelevant components inherent in time-varying motion data when the task outcome was categorical. We thus derived task-relevant and task-irrelevant components within the framework of linear classification methods, including logistic regression, as follows.

After estimating ***w***, we can obtain the task-relevant components by minimizing the following cost function

2 $${E}_{{\rm{rel}}}({{\boldsymbol{X}}}_{{\rm{rel}}})=\frac{1}{2}{({\boldsymbol{X}}{\boldsymbol{w}}-{{\boldsymbol{X}}}_{{\rm{rel}}}{\boldsymbol{w}})}^{T}({\boldsymbol{X}}{\boldsymbol{w}}-{{\boldsymbol{X}}}_{{\rm{rel}}}{\boldsymbol{w}})$$

for all trials because the task-relevant components provide the same classification probability as *p*(*d*_*t*_ = 1) = *f*(*w*_0_ + ***X***_*t*_***w***) = *f*(*w*_0_ + ***X***_rel,*t*_***w***). In other words, the task-relevant components provide all the task-relevant information included in the motion data. By solving $\frac{\partial {E}_{{\rm{rel}}}({{\boldsymbol{X}}}_{{\rm{rel}}})}{\partial {{\boldsymbol{X}}}_{{\rm{rel}}}}=0$ while avoiding the self-evident answer (i.e., ***X***_rel_ ≠ ***X***), we obtain

3 $${{\boldsymbol{X}}}_{{\rm{rel}}}={\boldsymbol{X}}\frac{{\boldsymbol{w}}{{\boldsymbol{w}}}^{T}}{| {\boldsymbol{w}}{| }^{2}},$$

where ∣***w***∣^2^ denotes the L2-norm of ∣***w***∣. The derived ${{\boldsymbol{X}}}_{{\rm{rel}}}={\boldsymbol{X}}\frac{{\boldsymbol{w}}{{\boldsymbol{w}}}^{T}}{| {\boldsymbol{w}}{| }^{2}}$ equaled ***X*** projected on the vector ***w*** because ***X***_rel_***w*** = ***Xw*** and ***X***_rel_***w***^⊥^ = 0 (***w***^⊥^ is a vector orthogonal to ***w***).

After obtaining ***X***_rel_, we can calculate the task-irrelevant components as follows:

4 $${{\boldsymbol{X}}}_{{\rm{irr}}}={\boldsymbol{X}}-{{\boldsymbol{X}}}_{{\rm{rel}}},$$

because ***X***_irr_***w*** = ***Xw*** − ***X***_rel_***w*** = 0. In other words, the task-irrelevant components do not provide any task-relevant information included in the motion data.

Notably, the above-mentioned derivations and interpretations of task-relevant and task-irrelevant components are the same as those used in our previous method, which focused on these components in continuous task outcomes^6^. Our data-driven technique thus forms a unified framework for extracting task-relevant and task-irrelevant motion components with either categorical or continuous task outcomes.

To investigate the properties of ***X***_rel_ and ***X***_irr_, we defined ${{\boldsymbol{X}}}_{{\rm{rel}},0}\in {{\bf{R}}}^{{T}_{0}\times D}$ as the task-relevant components in the trials estimated as throwing a fastball, where *T*_0_ indicates the number of these trials. Similarly, we defined ${{\boldsymbol{X}}}_{{\rm{rel}},1}\in {{\bf{R}}}^{{T}_{1}\times D}$, ${{\boldsymbol{X}}}_{{\rm{irr}},0}\in {{\bf{R}}}^{{T}_{0}\times D}$, and ${{\boldsymbol{X}}}_{{\rm{irr}},1}\in {{\bf{R}}}^{{T}_{1}\times D}$ (see the Supplementary Material for the details of the task-irrelevant components). Our method enabled an analysis of how the statistics of the components were modulated when subjects threw a fastball or breaking ball with or without the task requirement to use a similar throwing motion.

### Modulation of task-relevant components

Because the subjects were instructed to throw fastballs and breaking balls with a similar motion on the second day, we needed to evaluate the similarity between the throwing motions for fastballs and breaking balls. A possible and common measure of the similarity was the sensitivity index, or d’, previously proposed in the framework of signal detection theory.  d’ indicates the classification accuracy of two signals generated from different probability distributions. When *μ*_1_, *μ*_2_, and *σ* are the mean of the first distribution, that of the second distribution, and the common standard deviation of both distributions, d’ $=\,\frac{{\mu }_{1}-{\mu }_{2}}{\sigma }$. A smaller d’ indicates a more difficult classification; thus, d’ should be smaller when throwing a fastball and breaking ball using similar motions. There are two ways for d’ to become small: a small *μ*_1_ − *μ*_2_ or a large *σ*.

**Figure 3:**
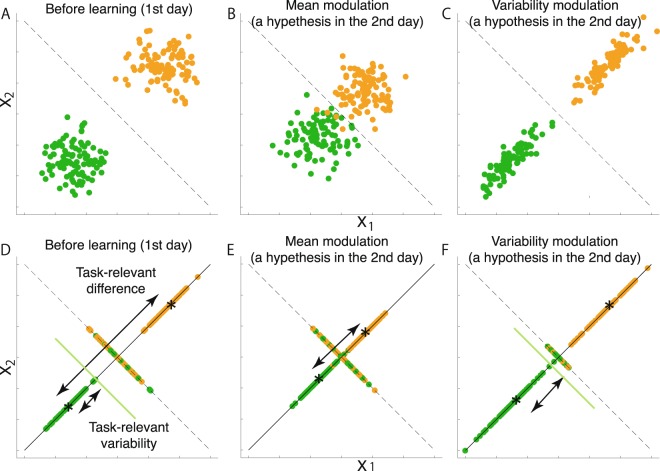
Simulated data. (**A**,**D**) Simulated data in a simple two-dimensional case ***X*** = (*X*_1_, *X*_2_). The green and orange dots indicate simulated data for different movement patterns (e.g., the green dots represent motion data for throwing a fastball, and the orange dots represent motion data for throwing a breaking ball). In this case, the classification boundary is drawn as a black dotted line. Panel (D) shows the task-relevant components (the green and orange dots on the solid black line) and the task-irrelevant components (the green and orange dots on the black dotted line) of the simulated data in panel (A). The task-relevant difference is defined as the distance between the means of task-relevant components, as indicated by the black asterisks. The task-relevant variability is defined as the variability in the task-relevant components, as shown by the variability between the black asterisk and the solid green line. The task-relevant variabilities can also be calculated for the data in a similar manner as denoted by orange dots. (**B**,**E**) A simulated mean modulation. In the mean-modulation hypothesis, the task-relevant differences are reduced, as shown in panel (E). (**C**,**F**) A simulated variability modulation. In the variability-modulation hypothesis, the task-relevant variance is increased, as shown in panel (F).

Two possibilities thus exist for the modulation of the task-relevant components depending on the task requirements. One possibility is modulation in the means of ***X***_rel,0_ and ***X***_rel,1_ (Fig. 3A,B,D,E). When subjects threw the fastballs and breaking balls in the trials where they had been instructed to use similar motion, the difference in the means of ***X***_rel,0_ and ***X***_rel,1_ should be reduced compared to the trials without the similar motion task requirement. We refer to this difference as the task-relevant difference. In addition, we refer to the possibility of the reduction of the task-relevant difference as mean modulation in the following. The second possibility is modulation in the variance of ***X***_rel,0_ and ***X***_rel,1_ (Fig. 3A,C–F); these variances could be increased with the task requirement, and this modulation enabled similar forms among conditions. We refer to the variance of ***X***_rel,0_ and ***X***_rel,1_ as task-relevant variability. In addition, we refer to the possibility of the increase of the task-relevant variability as variability modulation. We investigated these possibilities by conducting an experiment in which subjects threw fastballs or breaking balls.

On the first day, the subjects were instructed to throw either a fastball or a breaking ball towards a target. On the second day, the subjects were asked to throw the two types of balls with a similar form. No significant difference in ball trajectories and motion data was observed between the two days (see the Supplementary Materials).

**Figure 4:**
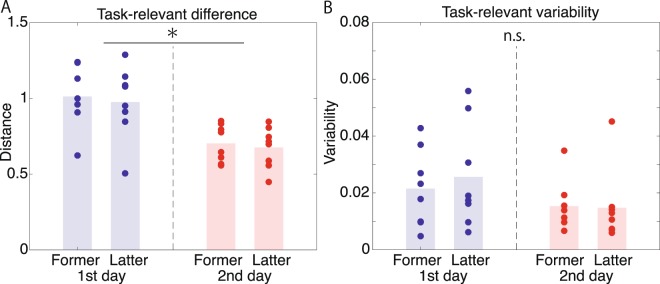
Experimental results. (**A**,**B**) The task-relevant difference and the variability calculated for the former and latter trials on the first and second day. A significant difference (denoted by a black asterisk) was observed only for the task-relevant difference between the first and the second day.

However, a significant modulation was observed in the task-relevant difference between the two days (Fig. 4A, *p* = 0.0029, paired t-test). In contrast to the task-relevant difference, there was no modulation of the task-relevant variability (Fig. 4B, *p* = 0.4777, paired t-test). The task-relevant variabilities were calculated by averaging the variance of ***X***_rel,0_ and that of ***X***_rel,1_. Although these results appeared to support mean modulation, there are two possibilities: the modulation may have been induced via learning or may have been produced due to the task requirement. If the first possibility were correct, we would expect to observe smaller task-relevant differences in the last 50 trials than in the first 50 trials within the first day. We divided the task-relevant components into those for the former and latter trials on each day. No significant interaction was observed between ‘Part’ and ‘Day’ (*p* = 0.890, see Methods for details), and no significant difference was observed in the task-relevant difference between the former and latter trials on the first day (*p* = 0.3276) or second day (*p* = 0.2667). These results contradicted the first possibility; therefore, the modulation of the task-relevant difference was not induced by learning within each day. In contrast, a significant difference was observed in the task-relevant difference between the former trials on the first day and second day (*p* = 0.00130) and between the latter trials on the first day and second day (*p* = 0.01001). Therefore, the task requirement to throw the fastballs and breaking balls with similar forms induced the mean modulation.

## Discussion

We proposed a data-driven method to detect task-relevant and task-irrelevant components when task outcomes are categorical using a machine learning technique. Our data-driven method can be applied even when the relationship between the motion data and the outcome is unknown; the relation can be estimated in a data-driven manner. Because the mathematical form between the current method and our earlier method focusing on continuous outcome^5,6^ is invariant, our data-driven approach forms a unified approach for detecting task-relevant and task-irrelevant motion components independent of the properties of the outcome. Our approach detected mean modulation in the task-relevant difference under the task requirement to throw fastballs and breaking balls with similar forms (Fig. 4A). The task requirement thus affected the mean rather than the variability in task-relevant motion components.

Although we relied on simple logistic regression, more complicated methods, such as mixture models^12–15^, kernel techniques^16^, and deep learning^17^, could also be applied. Linear regression has several advantages, including that it is related to motor primitive, a conventional model of motor control and learning^18–23^. Although most conventional models of motor learning focus on continuous outcome values, our method can also be applied to motor learning with categorical outcomes.

One limitation inherent to our method is that we assume a linearly separable case in the current study. When nonlinear transformation of ***X*** is indispensable for classification, kernel methods can work in many cases^12^. Using a kernel method, it is impossible to quantify the task-relevant differences except in the case of a linear kernel. The kernel method relies on nonlinear transformations of ***X***, *ϕ*(***X***). Although the explicit function of *ϕ*(***X***) is not necessary, the second-order statistics of *ϕ*(***X***) (i.e., kernel $K({\boldsymbol{X}},{{\boldsymbol{X}}}^{{\prime} })=\phi (X)\phi {({{\boldsymbol{X}}}^{{\prime} })}^{T}$) should be defined. Thus, we can possibly evaluate task-relevant variabilities (i.e., the second-order statistics) using $K({\boldsymbol{X}},{{\boldsymbol{X}}}^{{\prime} })$ but cannot evaluate task-relevant differences (i.e., first-order statistics). Because the data in the current study were linearly separable without nonlinear transformation of ***X*** (Fig. 2), we were able to quantify the modulation of task-relevant differences (Fig. 4).

Because the current study is the first step to apply a data-driven approach for discussing categorical motion outcomes, it is still unclear whether the current data-driven method is useful in other cases. The present study validated the effectiveness of a data-driven approach to discussing the throwing motions of fastball and breaking ball in naive participants. It thus remains unclear whether our method is practical in other contexts, such as the throwing motions of curveball and slider in naive participants, neurological patients, expert baseball pitchers, or baseball pitchers with a shoulder injury. In these and other contexts, some context-dependent modifications of a data-driven approach are possibly necessary and practical. To propose effective data-driven approaches to discuss diverse situations, such as detecting success-relevant components, patient-specific components, or professional-specific components, would be not only open questions but also promising future works.

One alternative to our method is to use Fisher linear discriminant analysis (LDA)^12^. Because LDA is a linear classification method whose classification performance is almost equivalent to logistic regression^24^, Eqs. (3) and (4) can still be applied. LDA could thus be a possible alternative to logistic regression. Other nonlinear classification techniques, such as quadratic discriminant analysis (QDA), are also possible alternatives to logistic regression when sufficient data are available. Given a sufficient quantity of data, nonlinear classification techniques can provide better classification accuracy than linear classification techniques. However, Eqs. (3) and (4) can be applied only to linear classification methods. To discuss task-relevant and task-irrelevant components, we must rely on linear classification methods, such as logistic regression or LDA.

It is possible to apply our method to some neural network models with some constraints. In general, a neural network model with *L* layers can be written as *y* = *g*(*h*(***XW***)), where *g* and *h* denote the nonlinear functions generated from the second to the *L*th layer and the nonlinear function in the first layer, respectively. In this form, we can still expect to be able to apply Eqs. (3) and (4) to the neural network. When *h* is a rectified linear function (*h*(*x*) = *ax* when *x* > *c* and *h*(*x*) = 0 otherwise, where *c* is a threshold), which is a common nonlinear function used in recent deep neural network models, many patterns of ***XW*** will generate identical 0 values. Because we assumed a one-to-one relation between ***X*** and *d* in Eq. (2), it cannot be appropriate for deriving task-relevant components under the rectified linear function. In contrast, when *h* is a sigmoid function, which is a common nonlinear function used in conventional neural network models, Eqs. (3) and (4) can be applied to extract task-relevant and task-irrelevant components.

Although it is difficult to define the relation between time-varying motion data and categorical task outcomes using mathematical equations such as parabolas, UCM focuses on kinematic parameters. UCM can thus be an alternative to our data-driven method in the current situation. A key point in UCM is that it requires linear approximations around averaged kinematics. For example, to apply UCM to our data, we need to evaluate the task-relevant and task-irrelevant components around averaged joint angles. In the simple case shown in Fig. 3, the averaged joint angles can be located in a blank region without any motion data. In addition, the linear approximation assumes that all the data are located close to the averaged values. In discussing the task-relevant and task-irrelevant components, this assumption will be invalid in several situations, such as in Fig. 3.

Another possible use case for UCM is to apply it separately to the motion data for throwing fastballs and breaking balls. Although this analysis is possible practically, it is beyond the assumption of the linear approximation theoretically. The linear approximation enables calculation of the task-relevant and task-irrelevant components around averaged joint angles by projecting the motion data onto the vectors corresponding to the directions that affect kinematic performance and to the directions that do not affect the performance, respectively. In addition, the directions of the vectors depend on the averaged joint angles for the linear approximation. Consequently, to analyze the current data separately in each condition, we need to compare task-relevant components using different vectors for each condition. For an imaginary and simplified example, the task-relevant directions can be proportional to 2*θ*_e_ + *θ*_s_ when throwing a fastball,and proportional to *θ*_e_ + 2*θ*_s_ when throwing a breaking ball, where *θ*_e_ and *θ*_s_ are the elbow and shoulder joint angles, respectively. Even when the mean and variance of the task-relevant components differ among the two conditions, it is difficult to judge whether the difference originates from the difference of task condition or task-relevant directions. In sum, although the UCM is sophisticated for discussing task-relevant and task-irrelevant components within the same condition, it is unsuitable for comparing those components, especially in situations that have multiple and categorical task outcomes.

## Supplementary information

Supplementary Information.

## Data Availability

The datasets analyzed in the current study are available from the corresponding author upon reasonable request.
