# Supplementary material for: A data-driven approach to decompose motion data into task-relevant and task-irrelevant components in categorical outcome
Source: Sci Rep. 2020 Feb 12;10:2422. doi: 10.1038/s41598-020-59257-z (PMC7015904; doi:10.1038/s41598-020-59257-z)
Supplement: Supplementary file 1 — Supplementary Information. [file 41598_2020_59257_MOESM1_ESM.pdf]

Supplementary materials for "A data-driven approach to decompose motion data into task-relevant and task-irrelevant components in categorical outcome"

Daisuke Furuki[1], **Ken Takiyama**[1]
[1]Department of Electrical and Electronic Engineering, Tokyo University of Agriculture and Technology, Koganei-shi, Tokyo 184-8588, Japan, Correspondence should be addressed to K.T. (t.j.ken.takiyama@gmail.com).

1 Statistical tests

Repeated measures analysis of variance (ANOVA) was conducted when there were no mention of the statistical tests, followed by Tukey's post hoc comparisons. The current study considered two within-subject factors, including 'Part' (the earlier or later performances within the same day) and 'Day' (day 1 or 2). All the statistical analyses were performed using MATLAB 2016a.

2 The mean and variance of ball trajectories and joint angles

Fig. S1A shows the difference in mean ball trajectory when throwing fastballs and breaking balls. The mean was calculated by averaging across all time frames. No significant difference was observed between the first and the second day (p=0.382, paired t-test). Fig. S1B shows the variability of the ball trajectories on the first and second days. The variability was calculated by averaging the variability of the ball trajectories for all time frames when throwing fastballs and breaking balls. No significant difference was observed between the first and second days (p=0.562, paired t-test).

Fig. S1C shows the difference in mean joint angles when throwing fastballs and breaking balls. The mean was calculated by averaging across all the joint angles and all time frames. No significant difference was observed between the first and second days (p=0.2504, paired t-test). Fig. S1D shows the variability of joint angles on the first and second days. The variability was calculated by averaging the variability of all the joint angles and all time frames when throwing fastballs and breaking balls. No significant difference was observed between the first and second days (p=0.109, paired t-test).

3 Task-irrelevant components

We refer to the difference between the mean of $\boldsymbol{X}_{\mathrm{irr},0}$ (the task-irrelevant components in the motion data classified as throwing a fastball) and that of $\boldsymbol{X}_{\mathrm{irr},1}$ (the task-irrelevant components in the motion data classified as throwing a breaking ball) as the task-irrelevant difference, and we refer to the variance of

$\boldsymbol{X}_{\mathrm{irr},0}$ and $\boldsymbol{X}_{\mathrm{irr},1}$ as the task-irrelevant variability. Fig. S1E shows the task-irrelevant difference and variability in a simulated two-dimensional case.

Although no significant modulation was observed in the task-irrelevant difference between the first and second days (Fig. S1F, $p = 0.4936$, paired t-test), a significant difference in task-irrelevant variabilities was observed (Fig. S1G, $p=0.000348$, paired t-test). No significant interaction between 'Part' and 'Day' was observed in the task-irrelevant variability ($p=0.290$). In terms of the task-irrelevant variabilities, a significant difference was observed between the former and latter trials on the first day ($p=0.0190$), but no significant difference was observed between those trials on the second day ($p=0.487$). Additionally, a significant difference was observed between the latter trials on the first day and the latter trials on the second day ($p=0.00962$), but no significant difference was observed between the former trials on the first day and the former trials on the second day ($p=0.0775$). Thus, in contrast to the task-relevant difference (Fig. 1G in the main manuscript), there was a possibility that the modulation of the task-irrelevant variability was induced not via the task requirement but by learning within a single day. Additionally, the task requirement induced an increase in task-irrelevant variability because the variability was larger on the second day than on the first day.

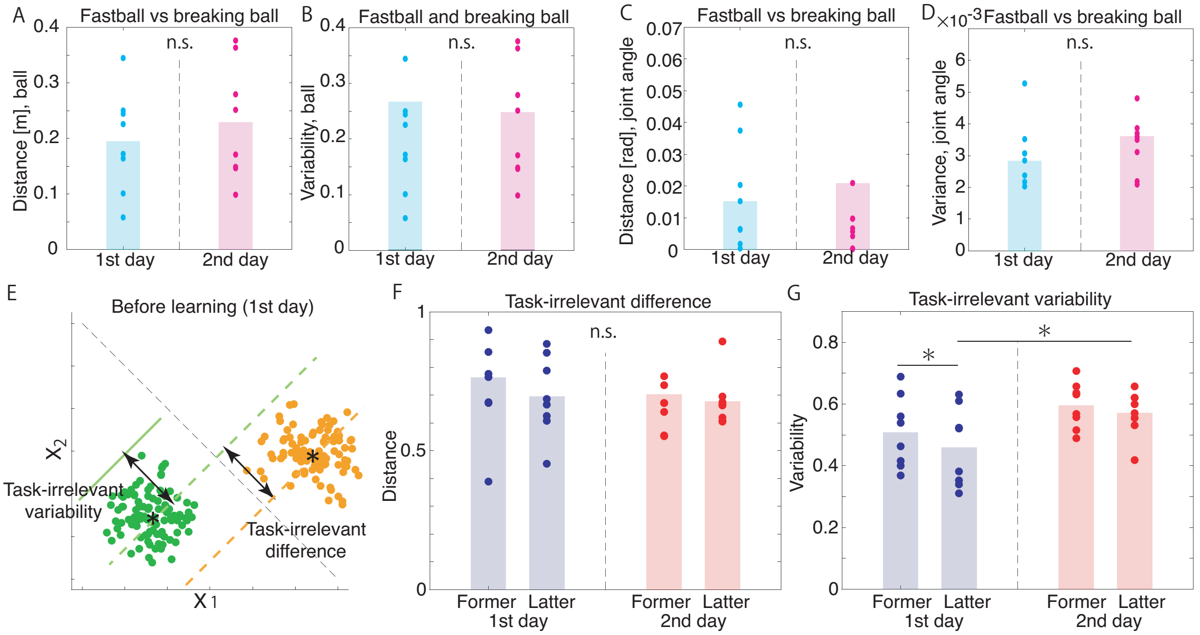

Fig. S1 Statistics of the ball trajectories, joint angles, and task-irrelevant components. (A) the difference in mean ball trajectory when throwing fastballs and breaking balls; (B) the variability of the ball trajectories; (C) the difference in mean joint angles when throwing fastballs and breaking balls; (D) the variability of the joint angles; (E) task-irrelevant difference and variability in a simulated two-dimensional case. In this example, the task-irrelevant components were calculated along with the classification boundary, in contrast to the task-relevant difference and variability (see Fig. 2 in the main manuscript for details). The task-irrelevant difference corresponds to the difference between the mean of the data denoted by green dots and that of the data indicated by orange dots, along with the black dotted line. The means are indicated by black asterisks, and the task-irrelevant difference corresponds to the distance between the green and orange dotted lines. The task-irrelevant variability in the data, denoted by the green dots, can be calculated as the variability along with the black dotted line, which corresponds to the variability between the black asterisks and the solid green line. The task-irrelevant variability in the data, denoted by the orange dots, can be calculated similarly. (F): Task-irrelevant difference on the first and second days. (G): Task-irrelevant variability on the first and second days. The black asterisks indicate significant differences.
